# Society Meetings

**Published:** 1896-10

**Authors:** 


### Society Meetings.

BUFFALO ACADEMY OF MEDICINE.—The following is the program, session of 1896-1897, of the section on obstetrics and gynecology :

*September 22, 1896.*—Post-partum hemorrhage: Etiology, pathology and diagnosis, Dr. P. W. Van Peyma ; treatment, Dr. R. L. Banta.

*October 27, 1896.*—Mammary inflammations : Etiology, pathology and diagnosis, Dr. W. G. Taylor ; treatment, Dr. Eugene Smith.

*November 24, 1896.*—Albuminuria of pregnancy : Etiology and pathology, Dr. A. A. Jones ; treatment, Dr. Henry R. Hopkins.

*December 22, 1896.*—Uterine displacements : Etiology and pathology, Dr. M. A. Crockett ; diagnosis and treatment, Dr. Stephen Y. Howell.

*January 26, 1897.*—Extrauterine pregnancy : Etiology, pathology and diagnosis, Dr. Marcell Hartwig ; treatment, Dr. H. E. Hayd.

*February 23, 1897.*—Drainage following abdominal operations : Dr. Chauncey P. Smith ; treatment of shock and collapse following operations, Dr. John Parmenter.

*March 23, 1897.*—Disorders of menstruation : Etiology and pathology, Dr. Lillian Craig Randall ; diagnosis and treatment, Dr. H. D. Ingraham. The relation of atrophic rhinitis in young women to disorders of menstruation, Dr. John E. Bacon.

*April 27, 1897.*—Abortion : Etiology and pathology, Dr. C. S. Jewett ; diagnosis and treatment, Dr. C. C. Frederick. A plea for uterine curretage as a routine measure after abortion before the third month, Dr. S. Goldberg.

*May 25, 1897.*—Subject to be announced, Dr. Irving Snow. Infantile dyspepsia, Dr. A. L. Benedict.

*June 22, 1897.*—(The section entertains the academy.) Puerperal septicemia: Etiology and pathology, Dr. L. G. Hanley; diagnosis and treatment, Dr. M. D. Mann.

Meetings commence promptly at 8.45 P. M.

DR. H. E. HAYD, *President*,
493 Delaware avenue.
DR. WALTER S. REYNOLDS, *Secretary*,
381 Hampshire street.

---

THE following is the complete list of officers and committees of the Medical Society of the State of New York: President, Dr. James D. Spencer, Watertown; vice-president, Dr. L. Duncan Bulkley, New York; secretary, Dr. Frederick C. Curtis, 17 Washington avenue, Albany; treasurer, Dr. Charles H. Porter, 103 Lancaster street, Albany.

Business Committee—Drs. Seneca D. Powell, chairman, 12 W. 40th street, New York; Willis G. Macdonald, 27 Eagle street, Albany; Ernest Wende, 471 Delaware avenue, Buffalo.

Committee of Arrangements—Drs. Wm. J. Nellis, 210 State street, Albany; Wm. Hailes, Albany; Reynold W. Wilcox, New York.

Committee on By-laws—Drs. H. D. Wey, Elmira; W. J. Herriman, Rochester; F. C. Curtis, Albany.

Committee on Hygiene—Drs. Henry R. Hopkins, 433 Franklin street, Buffalo; Lewis S. Pilcher, Brooklyn; Daniel Lewis, New York; Willis G. Macdonald, Albany; O. W. Peck, Oneonta; Lucien Howe, Buffalo; A. M. Campbell, Mt. Vernon.

Committee on Legislation —Drs. A. Walter Suiter, Herkimer; M. J. Lewi, New York; J. M. Winfield, Brooklyn.

Committee on Ethics—Drs. Charles Jewett, 330 Clinton avenue, Brooklyn; Eugene Beach, Gloversville; John L. Heffron, Syracuse.

Committee on Prize Essays—Drs. Abraham Jacobi, 110 W. 34th street, New York; Henry Hun, Albany; Wm. F. Cheesman, Auburn.

Committee of Publication—Drs. F. C. Curtis, Albany; F. D. Bailey, Brooklyn; M. D. Mann, Buffalo; Chas. H. Porter, Albany.

The next annual meeting of the society will be held in Albany, N. Y., January 26–28, 1897.

---

THE twenty-ninth annual meeting of the Medical Association of Central New York will be held in Rochester, Tuesday, October

20, 1896. The titles of papers to be presented should be sent the secretary by October 1st to be included in the program. To insure prompt publication of all papers and discussions in the transactions of the association the manuscript should be given to the secretary at the meeting. As these papers are to appear first in the BUFFALO MEDICAL JOURNAL, they must not have been published in any other journal or the secretary cannot undertake to insure their appearance in the transactions. Every member paying the annual dues of one dollar is entitled to a copy of the transactions. C. A. Vanderbeek, secretary, 113 North street, Rochester, N. Y.

---

THE election of officers of the American Microscopical Society, held at Pittsburg, Pa., August 18–20, 1896, resulted as follows: President, Professor E. W. Claypole, Akron, O.; vice-president, C. C. Mellor, Pittsburg, Pa.; second vice-president, Dr. A. M. Bleile, Columbus, O.; secretary, Dr. Wm. C. Krauss, Buffalo, N. Y.; treasurer, Magnus Pflaum, Pittsburg, Pa.; executive committee, Dr. A. A. Young, Newark, N. Y.; Mrs. S. P. Gage, Ithaca, N. Y.; Dr. W. P. Manton, Detroit, Mich.

---

THE annual election of officers of the Association of Erie Railway Surgeons, held at Lakewood, N. Y., September 21, 1896, resulted as follows: President, Dr. Webb J. Kelly, Galion, O.; vice-president, Dr. F. W. Thomas, Marion, O.; secretary and treasurer, Dr. W. W. Appley, Cohocton; executive committee, Dr. C. M. Daniels, Buffalo; Dr. C. S. Parkhill, Hornellsville; Dr. L. H. Leyman, Huntington, Ind.

---

THE election of officers of the Mississippi Valley Medical Association, held at St. Paul, Minn., September 15–18, 1896, resulted as follows: President, Dr. T. H. Stuckey, Louisville, Ky.; first vice-president, Dr. C. A. Wheaton, St. Paul, Minn.; second vice-president, Dr. Paul Paquin, St. Louis, Mo.; and Dr. H. W. Loeb, secretary. The next meeting will be held at Louisville, Ky., in October, 1897.

---

THE annual meeting of the New York State Association of Railway Surgeons, under presidency of C. S. Parkhill, M. D., of Hornellsville, will be held on November 17, 1896, at the Academy of Medicine, New York City, C. B. Herrick, M. D., Troy, N. Y., Secretary.

Program of the Tri-state Medical Society, to be held at Chattanooga, Tenn., Tuesday, Wednesday and Thursday, October 13, 14 and 15, 1896 : 1. Convulsions in children, treated with large doses of morphine, Y. L. Abernathy, Hill City, Tenn. ; to open the discussion, P. D. Sims, G. Manning Ellis. 2. The theory of antipyretics, P. L. Brouillette, Huntsville, Ala. ; to open the discussion, W. C. Bilbro, J. W. Duncan, J. B. Cowan. 3. Cystitis ; report of cases, D. S. Middleton, Rising Fawn, Ga. ; to open discussion, G. A. Baxter, George S. Brown. 4. A new splint for fractures of the humerus below the surgical neck, G. A. Baxter, Chattanooga, Tenn. ; to open the discussion, C. Holtzclaw, W. F. Westmoreland. 5. Humphrey's operation (amputation of the penis), with presentation of patient, C. Holtzclaw, Chattanooga, Tenn. ; to open the discussion, Duncan Eve, Y. L. Abernathy. 6. Diseases of the veru montanum (caput gallinaginis), W. Frank Glenn, Nashville, Tenn. ; to open the discussion, Wm. S. Goldsmith, J. A. Goggans. 7. Operations for abscesses of the liver, W. C. Towns, Chattanooga, Tenn. ; to open the discussion, W. E. Davis, Richard Douglas, W. D. Haggard, Jr. 8. Acute pelvic congestion, Valentine Taliaferro, Atlanta, Ga.; to open the discussion, W. G. Bogart, George R. West. 9. A few unique cases of abdominal section, J. A. Goggans, Alexander City, Ala. ; to open the discussion, R. M. Cunningham, Richard Douglas. 10. Treatment of pus in the pelvis, W. E. B. Davis, Birmingham, Ala. ; to open the discussion, R. J. Trippe, J. B. S. Holmes. 11. Vaginal hysterectomy for bilateral suppurative processes of the uterine adnexa, W. D. Haggard, Jr., Nashville, Tenn.; to open the discussion, Richard Douglas, J. A. Goggans. 12. Some remarks on syphilis, W. F. Westmoreland, Atlanta, Ga. ; to open the discussion, Richard Douglas, W. Frank Glenn. 13. Observations on the treatment of specific and nonspecific venereal ulcers, Wm. S. Goldsmith, Atlanta, Ga. ; to open the discussion, Geo. S. Brown, W. Frank Glenn. 14. Bacteriological data in the drainage of the peritoneal cavity, Geo. S. Brown, Birmingham, Ala.; to open the discussion, R. M. Cunningham, W. F. Westmoreland. 15. A statistical report of some of the more recent remedies used in the treatment of tuberculosis and summary of recent preventative methods of value, R. H. Hayes, Union Springs, Ala. ; to open the discussion, R. M. Cunningham, H. Berlin, J. C. LeGrand. 16. The Turkish bath ; its therapeutic indications, Louise Eleanor Smith, Chattanooga, Tenn. ; to open the discussion, E. A. Cobleigh, Katherine R. Collins. 17. Microscopical and chemical aids to diagnosis, Katherine R. Collins, Atlanta, Ga.; to open the discussion, Louis Eleanor Smith, W. C. Townes, G. W. Drake. 18. Some obstetrical complications, with report of cases, R. R. Kime, Atlanta, Ga.; to open the discussion, W. G. Bogart, Richard Douglas, W. D. Haggard, Jr. 19. Puerperal eclampsia, Seale Harris, Union Springs, Ala.; 20. Puerperal eclampsia, J. E. George, Rockwood, Tenn.; to open the discussion, R. R. Kime, R. M. Cunningham. 21. Medicine—hippocratic and operatic, John P. Stewart, Attalla, Ala.; to open the discussion, J. B. Cowan, Y. L. Abernathy. 22. Diseases and treatment of the accessory sinuses of the nose, B. F. Travis, Chattanooga, Tenn.; to open the discussion, N. C. Steele, G. C. Savage. 23. Subject to be announced, G. C. Savage, Nashville, Tenn.; to open the discussion, B. F. Travis, J. M. Crawford. 24. Paper on diseases of the eye, Alec. Sterling, Atlanta, Ga.; to open the discussion, G. C. Savage, B. F. Travis. 25. Paper on diseases of the eye, J. M. Crawford, Atlanta, Ga.; to open the discussion, Alec. Sterling, T. Hilliard Wood. 26. Report of a case of bradycardia, W. C. Bilbro, Murfreesburg, Tenn.; to open the discussion, J. R. Rathmell, J. W. Duncan. 27. The Woodbridge treatment of typhoid fever, J. W. Duncan; to open the discussion, J. B. Murfree, Jno. P. Stewart. 28. Treatment of cancer of the skin, C. R. Achison, Nashville, Tenn.; to open the discussion, E. A. Cobleigh, M. B. Hutchins. 29. President's address—The doctor of medicine, J. B. Murfree, Murfreesboro, Tenn. Dr. Frank T. Smith, secretary, Chattanooga, Tenn.

---

Transportation Arrangement for the Mexican Meeting of the Pan-American Medical Congress.—Dr. H. L. E. Johnson, 1400 L street, N. W., Washington, D. C., has been elected chairman of the special committee on transportation. All communications relative to rates, reservation in the regular trains, etc., should be addressed to him. A rate of one fare for the round trip has been secured between St. Louis, New Orleans, and all other trans-Mississippi points and the City of Mexico. It is confidently expected that this rate will be extended over the entire territory of the United States. Arrangements are in progress for a splendidly-equipped train of sleeping and observation cars, with first-class dining-car service. Dr. Johnson will presently be in position to announce a rate, which will include railroad fare, sleeping and dining-car service both ways, and in the City of Mexico, and covering the expenses of various side trips to most important historic points in the Republic. Dr. Charles A. L. Reed, secretary international executive committee.

